# Predicting factors for primary cervical cancer recurrence after definitive radiation therapy

**DOI:** 10.1259/bjro.20210050

**Published:** 2021-09-19

**Authors:** Mitsuru Okubo, Tomohiro Itonaga, Tatsuhiko Saito, Sachika Shiraishi, Daisuke Yunaiyama, Ryuji Mikami, Akira Sakurada, Shinji Sugahara, Koichi Tokuuye, Kazuhiro Saito

**Affiliations:** ^1^ Department of Radiology, Tokyo Medical University Hospital, Tokyo, Japan

## Abstract

**Objectives:**

The study aimed to retrospectively investigate the apparent diffusion coefficient (ADC) of primary cervical cancer to examine the recurrence correlations in patients treated with radiotherapy (RT).

**Methods:**

The ADC of 31 patients with cervical cancer treated with RT were analyzed as possible risk factors for recurrence. A receiver operating characteristic (ROC) curve of the mean ADC (ADCmean) for the recurrence was generated to determine the cut-off value that yielded optimal sensitivity and specificity. The patient population was subdivided according to the risk factors for recurrence, and the disease-free survival (DFS) was analyzed. The following were investigated to explore the risk factors for recurrence: age, performance status, stage, pelvic lymph node metastasis, histologic tumor grade, maximal diameter of the primary tumor, chemotherapy, and ADCmean.

**Results:**

The median follow-up duration of the patients was 25 months. The recurrence was recognized in 9 (29%) of the 31 cases. The ROC analysis of recurrence showed that the area under the ADCmean curve was 0.889 (95% CI, 0.771–1.000; *p* = 0.001). The cut-off value of ADC mean was 0.900 × 10^− 3^ mm^2^/s, with a sensitivity of 86.4% and a specificity of 88.9%. By univariate analysis, the ADCmean was the only factor significantly associated with recurrence.

**Conclusion:**

The ADCmean of the primary tumor is a potential predictive factor for the recurrence in of cervical cancer.

**Advances in knowledge:**

The ADCmean of the primary tumor is a predictor of recurrence in patients with pre-treatment cervical cancer evaluation.

## Introduction

Cervical cancer is the second most common gynecological malignancy.^1^ The International Federation of Gynecology and Obstetrics (FIGO) reported that a 5 year recurrence rate of 28% and an overall mortality rate of 27.8% for females with cervical cancer.^2^ Depending on the FIGO stage and histological subtype, the primary treatment consists of surgery, radiotherapy (RT), chemotherapy, or concurrent chemoradiation therapy. RT consisting of external-beam RT (EBRT), cisplatin-based chemotherapy, and intracavitary brachytherapy is the recommended standard treatment for locally advanced cervical cancer.^3^ However, a substantial number of patients experience locoregional recurrence or distant metastasis despite treatment.^4,5^ Poor prognostic factors for cervical cancer include pelvic lymph node metastasis, parametrial involvement, positive surgical margins, large tumor diameter, deep stromal invasion and the presence of tumor in capillary lymphatic spaces.^6^ However, these parameters are not sufficient to accurately predict prognosis. It is now accepted that new approaches to cervical cancer are pivotal to improving this disease’s prognosis.

MRI has an essential role in diagnosing cervical cancer, particularly for local staging. Diffusion-weighted imaging (DWI) is a functional imaging technique that analyzes differences in extracellular water proton movement, allowing for discrimination between tissues with varying cellularity.^7^ Additionally, this technique allows quantification of diffusion by calculating the apparent diffusion coefficient (ADC) values. In malignant tumors, the increased cellular density restricts water diffusion in the interstitial space, thus, lowering the ADC. Some studies showed that low ADC values are related with recurrence and a poor survival rate,^8,9^ while some found low ADC values in patients with good treatment responses.^10,11^ Other studies concluded that there is insufficient evidence to use pre-treatment ADC to predict the treatment efficacy.^12–14^ Therefore, it has been suggested that the ADC may provide useful information on tumor cellularity, tumor aggressiveness, and subtype characterization.^15–18^

In this study, we investigated the ADC of primary squamous cell cervical cancer to examine its correlation to recurrence in patients treated with RT.

## Methods and materials

### Study design and patients

From May 2012 to December 2019, 41 consecutive patients with pathologically diagnosed squamous cell uterine cervical cancer were treated with definitive RT at Tokyo Medical University Hachioji Medical Center. All patients provided written informed consent, and the Ethical Review Board approved this study of the authors' institution. Of the 41 patients, 31 patients who underwent MRI taken by the same machine within 30 days prior to the start of treatment were selected in this retrospective analysis. No patients enrolled in this study received any neoadjuvant chemotherapy before RT.

### Treatment

Three-dimensional conformal RT was planned and performed with the patient in the supine position. For treatment planning, all patients underwent pelvic CT at a 2.5 mm slice thickness. Typically, the patients underwent EBRT with a photon beam of 10 MV. RT consisted of a combination of whole pelvic (WP) EBRT and high-dose-rate intracavitary brachytherapy (HDR-ICBT). WP-EBRT was delivered for 5 days during a week to achieve a total dose of 50.4 Gy/28 fractions. The WP-EBRT was initially delivered without a midline block (MB) using a box technique. Subsequently, the next phase of WP-EBRT was administered through the same WP field with a MB width of 3 or 4 cm using anteroposterior opposite ports. The first HDR-ICBT was performed after the MB insertion. HDR-ICBT was performed once a week with a fraction dose of 6 Gy prescribed at point A using Ir-192 afterloading machines. HDR-ICBT was not allowed on the same day as the EBRT. The relationship between RT schedule and patient’s stage was shown in Table 1. The cumulative linear quadratic equivalent doses (EQD2)^19^ at point A, which were the summation of the EBRT doses without the MB and HDR-ICBT doses. For patients who had an inadequate response to EBRT or failed tandem insertion, additional WP-EBRT without the MB was allowed to a total dose of 50.4 Gy. The total HDR-ICBT dose was 12 Gy per 2 fractions at point A.

**Table 1.** The relationship between RT schedule and patients’ stage

EBRT		HDR-ICBT	Total EQD2 at point A	Patients’ stage
WP	WP (MB)			
30.6 Gy/17 Fr	19.8 Gy/11 Fr	24 Gy/4 Fr	62 Gy	Stage Ib/IIb
39.6 Gy/22 Fr	10.8 Gy/6 Fr	18 Gy/3 Fr	63 Gy	Stage IIIa/IVA
50.4 Gy/28 Fr	0 y	12 Gy/2 Fr	66 Gy	

EBRT, external-beam radiotherapy; EQD2, equivalent dose in 2 Gy per fraction; HDR-ICBT, high-dose-rate intracavitary brachytherapy; MB, midline block; WP, whole pelvic radiotherapy.

Weekly cisplatin at a dose of 40 mg/m^2^ was administered for five courses during the RT period. Of the 31 patients, 26 (84%) received concurrent cisplatin chemotherapy, however the remaining 5 (16%) patients did not receive concurrent chemotherapy due to the low stage or the presence of comorbidities.

### MRI technique and image analysis

**Figure 1.**
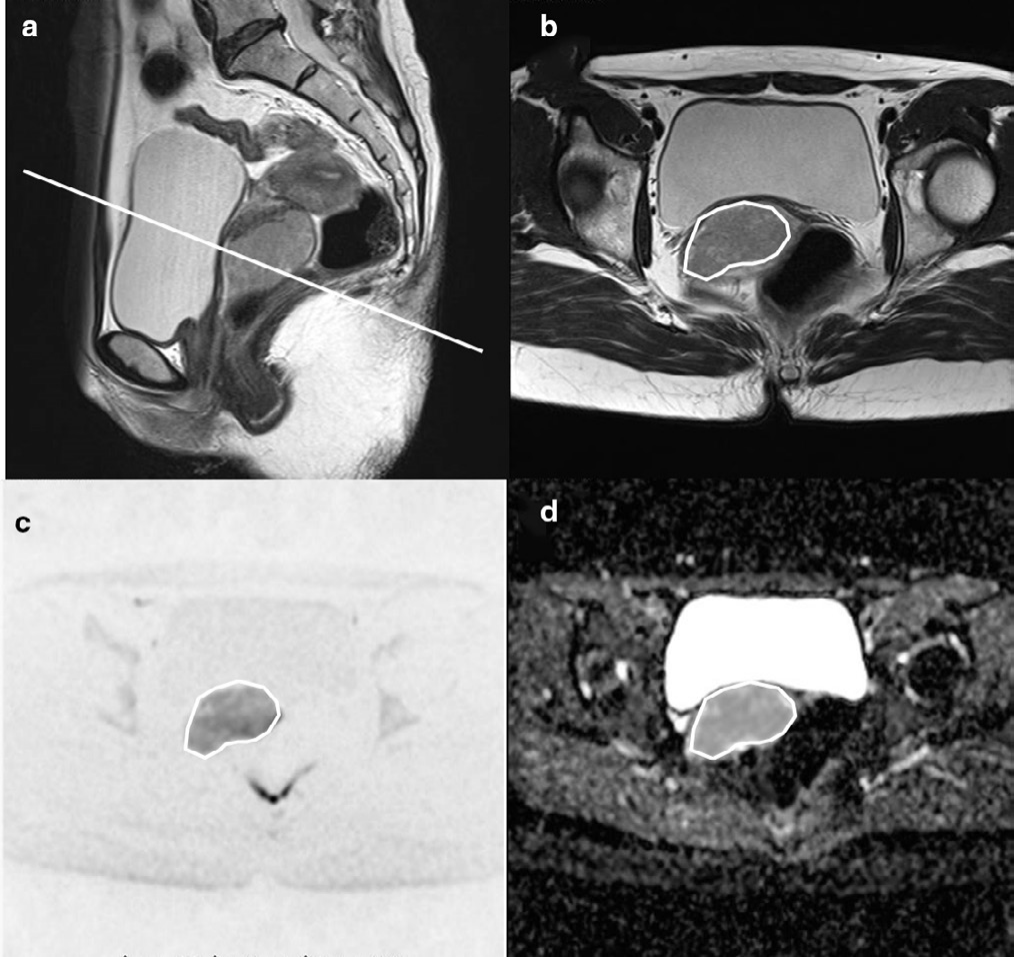
A typical ROI placement for tumors. (a) *T*_2_ weighted sagittal image. The white line is the reference line for the axial image on (b–d.) Axial image for *T*_2_ weighted and DW image, ADC map. ROIs were drawn manually along the edge of the lesions to cover as much tumor area as possible on a slice of the largest tumor area without excluding cystic or necrotic areas. ADC, apparent diffusion coefficient; DWI, diffusion-weighted imaging; ROI, region of interest

MRI was performed using a 1.5 T MR system (Magnetom Avanto; Siemens, Erlangen, Germany) with a 6-channel phased-array coil. Routine pelvic MRIs were acquired as follows: sagittal *T*_1_ weighted fast spin-echo (FSE) images [repetition time (TR)/echo time (TE), 550/11 ms; flip angle, 180°; section thickness/intersection gap, 4/0.4 mm; a field of view (FOV), 250 × 250; matrix size, 230 × 384; the number of excitation, 4], and axial, sagittal, and coronal *T*_2_ weighted FSE images [TR/TE, 4000/84 ms; flip angle, 150°; section thickness/intersection gap, 4/0.4 mm; FOV, 250 × 250; matrix size, 230 × 384; the number of excitation, 4]. Axial DW images were then obtained. Imaging parameters for DW imaging were as follows: TR/TE, 4000/75; flip angle, 90°; section thickness/intersection gap, 4/0.4 mm; FOV, 280 × 280 matrix size, 128 × 128; bandwidth, 2170 Hz/pixel; the number of excitation, 4, using a chemical shift-selective fat suppression technique. The corresponding b-values to the diffusion sensitizing gradient were 0 and 1000 s/mm^2^. The ADC values were calculated from the regions of interest (ROIs) by dividing the signal intensity by 1000 to obtain ADC values × 10^−3^ mm^2^/s. The ROI placements and ADC calculations were made in the tangible portions of the primary tumor’s maximum sectional diameter, avoiding cystic or necrotic portions. Polygonal ROIs were placed manually on the maximum axial section of the primary tumor on the ADC map. The mean ADC value (ADCmean) of all full pixels within the ROI was obtained. One radiation oncologist with 17 years of experience drew all ROIs referencing the *T*_2_ weighted images. A typical ROI placement for a tumor is shown in Figure 1.

### Evaluation of the local response and toxicity

Local response was estimated by physical examination at 1 month after completing RT. The regular follow-up visits were performed at 2–3 month intervals for the first 2 years, then every 4–6 months after that, in the absence of clinical symptoms. At each follow-up visit, evaluation consisted of medical history, physical examination, Papanicolaou smear, ultrasonography, CT scans, and tumor marker assessment. The toxicity data were collected retrospectively from patient files. In evaluating the acute or late effect, toxicity criteria of the Common Terminology Criteria for Adverse Events v. 3.0 was used.

### Statistical analyses

The endpoint was disease-free survival (DFS), which was defined as the time from the start of RT to clinical progression or death for any cause. Statistical analyses were performed using the Mann–Whitney *U* test to compare the recurrence and non-recurrence followed by Fisher’s protected least significance test for all pairwise comparisons. The ROC curve of the ADCmean for the recurrence was generated to determine the cut-off value that yielded optimal sensitivity and specificity. The patient population was subdivided according to the risk factors for recurrence. Moreover, the DFS was analyzed using the Kaplan–Meier method. The log-rank tests were used to examine the differences between the survival curves. The following were investigated: age, performance status, stage, pelvic lymph node metastasis, histologic tumor grade, maximal diameter of the primary tumor, concurrent cisplatin chemotherapy, and ADCmean to explore the risk factors for recurrence. Univariate logistic regression analyses were performed to evaluate the data using IBM SPSS Statistics 20.0 (SPSS, Armonk, NY). Multivariate analysis was not performed owing to the limited data. A two-sided *p*-value < 0.05 was considered statistically significant for all statistical tests.

## Results

### Patient outcomes

**Figure 2.**
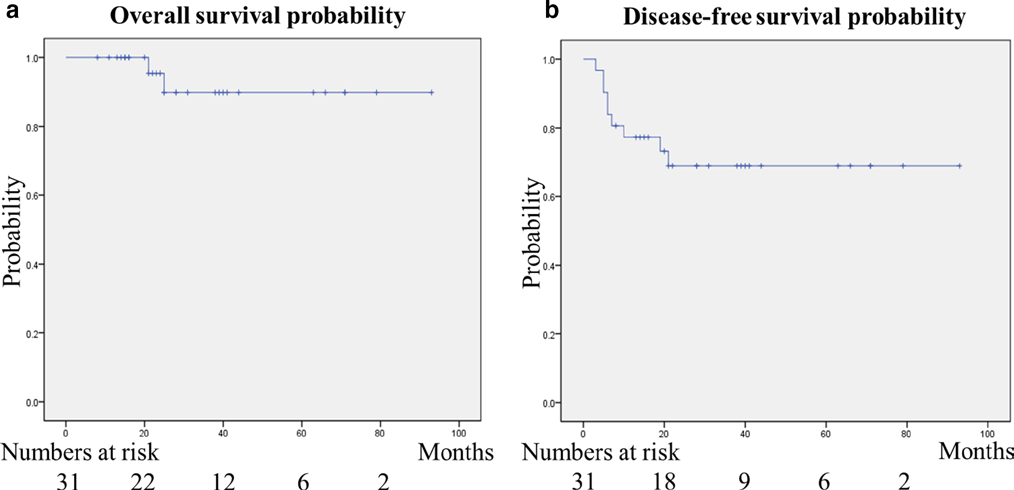
Overall survival and disease-free survival probabilities. (a) Overall survival probabilities at 1 and 3 years were 100 and 90%, respectively. Two (6%) were diagnosed identified as with a cancer-related death, at 21 and 25 months after the start of RT. (**b**) Disease-free survival probabilities at 1 and 3 years were 77 and 69%, respectively. The recurrence was recognized in 9 (29%) of the 31 cases.

The patients and tumor characteristics are presented in Table 2. The patients' median age was 62 (range, 25–87) years. Of the total, 94% of patients had Eastern Cooperative Oncology Group (ECOG) performance status of 0 or 1. The FIGO system defined the patients’ stage as follows: three stage Ib1 cancers; one stage Ib2 cancers; two stage IIa1 cancers; 15 stage IIb cancers; one stage IIIa cancers; six stage IIIb cancer; and, three stage IVa cancers. Three patients (10%) had double cancers. The patients' median follow-up duration was 25 months (range, 8–93). Overall survival probabilities at 1 and 3 years were 100 and 90%, respectively (Figure 2a). Two (6%) patients were identified with a cancer-related death at 21 and 25 months after RT. DFS probabilities at 1 and 3 years were 77 and 69%, respectively (Figure 2b). The recurrence was recognized in 9 (29%) of the 31 cases; local failure only in 3 cases, and local failure and distant metastasis in 2, distant metastasis only in 4. The median time for recurrence was 6 months (range, 3–21) after RT initiation.

**Table 2.** Patient and tumor characteristics

No. of patients	31
Age (years), median (range)	62 (25–87)
Performance status	
0	27
1	2
2	2
FIGO stage	
Ib1	3 (10)
Ib2	1 (3)
IIa1	2 (6)
IIb	15 (48)
IIIa	1 (3)
IIIb	6 (19)
IVa	3 (10)
Pelvic lymph node metastasis (percentage)	
Yes	15 (48)
No	16 (52)
Histologic tumor grade (percentage)	
Well differentiated	2 (6)
Moderate differentiated	22 (71)
Poor differentiated	7 (23)
Size of primary tumor (percentage)	
≤4 cm	7 (23)
>4 cm	24 (77)
Chemotherapy (percentage)	
Yes	26 (84)
No	5 (16)

### Association between ADC mean and recurrence

**Figure 3.**
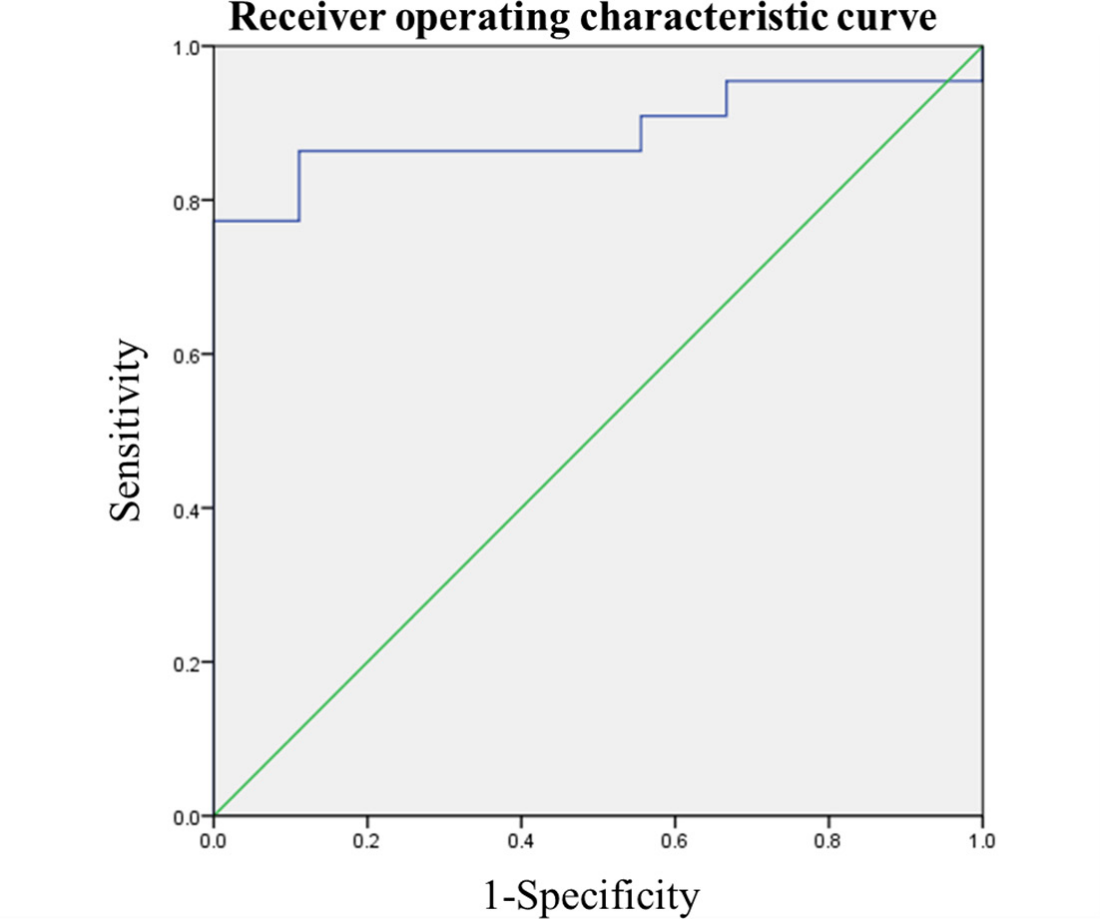
Receiver operating characteristic analysis of recurrence The area under the curve for ADCmean was 0.889 (95% CI, 0.771–1.000; *p* = 0.001). The cut-off value of ADCmean was 0.900 × 10^−3^ mm^2^/s, with a sensitivity of 86.4% and a specificity of 88.9%. ADC, apparent diffusion coefficient CI, confidence interval.

The average values of ADCmean for the primary tumor of cervical cancer with recurrence and non-recurrence were found to be 0.840 ± 0.064×10^−3^ mm^2^/s and 0.949 ± 0.082×10^−3^ mm^2^/s, respectively. The difference in ADCmean between the two groups was statistically significant (*p* < 0.001), calculated with the Mann–Whitney *U* test. ROC analysis of recurrence showed that the area under the ADCmean curve was 0.889 (95% CI, 0.771–1.000; *p* = 0.001) (Figure 3). The cut-off value of ADCmean was 0.900 × 10^− 3^ mm^2^/s, with a sensitivity of 86.4% and a specificity of 88.9%.

### Univariate analyses

**Figure 4.**
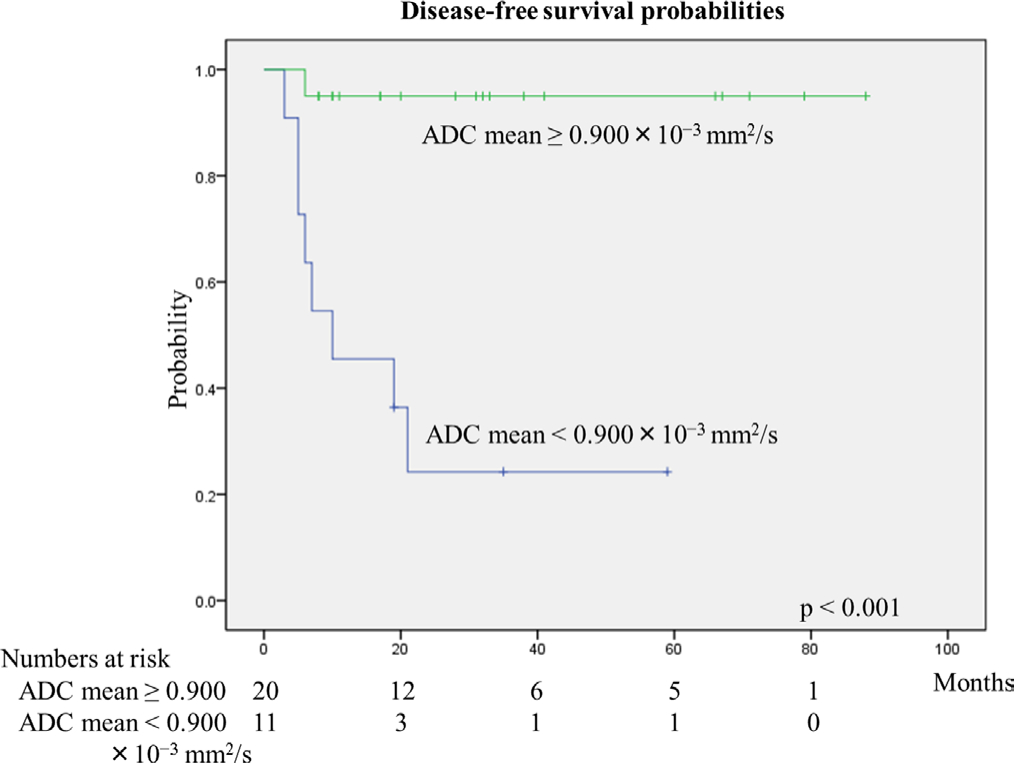
Disease-free survival probabilities associated with ADC mean The 2–year DFS probabilities for patients with cervical cancer of ADCmean ≥ 0.900×10^−3^ mm^2^/s and <0.900 ×10^−3^ mm^2^/s were 95 and 24%, respectively. DFS probability difference between these patients was calculated using the log–rank test and was found to be statistically significant (*p* < 0.001). ADC, apparent diffusion coefficient; DFS, disease-free survival

The relationships between the risk factors and recurrence are summarized in Table 3. By univariate analysis, the ADCmean was the only factor significantly associated with recurrence (*p* < 0.001), calculated with the log-rank test. The 2-year DFS probabilities for patients with cervical cancer of ADCmean ≥ 0.900×10^−3^ mm^2^/s and <0.900 ×10^−3^ mm^2^/s were 95 and 24%, respectively (Figure 4). The DFS probability between these patients was calculated using the log-rank test and was found to be statistically significant (*p* < 0.001). The relationships between the ADCmean and the other risk factors, calculated with the Mann–Whitney *U* test, are shown in Table 4. ADCmean had been not related with the other risk factors.

**Table 3.** Risk factors associated with recurrence

	Recurrence *n* = 9	*p*-value
Age (≤60 y vs. >60 y)	15% (2/13) *vs* 39% (7/18)	0.168
PS (0 vs. ≥1)	30% (8/27) *vs* 25% (1/4)	0.989
FIGO stage (Ib1/IIb *vs* IIIa/IVa)	33% (7/21) *vs* 20% (2/10)	0.484
Histologic tumor grade (well/moderate *vs* poor)	33% (8/24) *vs* 14% (1/7)	0.384
Pelvic lymph node metastasis (negative *vs* positive)	25% (4/16) *vs* 33% (5/15)	0.581
Maximal diameter of primary tumor (<4 cm vs. ≥4 cm)	29% (2/7) *vs* 29% (7/24)	0.893
Concurrent cisplatin chemotherapy (negative *vs* positive)	40% (2/5) *vs* 27% (7/26)	0.556
ADCmean × 10^−3^ mm^2^/s (<0.900 vs.≥0.900)	73% (8/11) *vs* 5% (1/20)	<0.001

ADC, apparent diffusion coefficient;FIGO, International Federation of Gynecology and Obstetrics; PS, performance status.

**Table 4.** Relationships between the ADC mean and the other risk factors

Median (range)	ADC mean × 10^−3^ mm^2^/s	*p*-value
Age		0.779
≤60 y	0.930 (0.692–1.018)	
>60 y	0.943 (0.726–1.069)	
PS		0.616
0	0.930 (0.692–1.069)	
≥1	0.955 (0.831–1.000)	
FIGO stage		0.597
Ib1/IIb	0.930 (0.692–1.018)	
IIIa/IVa	0.951 (0.726–1.069)	
Histologic tumor grade		0.171
Well/moderate	0.934 (0.692–1.069)	
Poor	0.980 (0.834–1.058)	
Pelvic lymph node metastasis		0.635
Negative	0.943 (0.692–1.069)	
Positive	0.930 (0.726–1.018)	
Maximal diameter of primary tumor		0.539
<4 cm	0.947 (0.692–1.069)	
≥4 cm	0.934 (0.726–1.058)	

ADC, apparent diffusion coefficient;PS, performance status.

### Complications

Table 5 shows the acute and late complications of irradiation. 5 (25%) of 31 patients had Grade 2 acute diarrhea. One patient had Grade 2 late proctitis, and the other had Grade 2 hematuria. No patients showed Grade 3 or greater acute and late toxicities. The clinical data and risk factors for all cases are shown in Table 6.

**Table 5.** Acute and late toxicities

Grade	0 or 1	2	3	4
Acute toxicities				
Cystitis	31	0	0	0
Diarrhea	26	5	0	0
Late toxicities				
Proctitis	30	1	0	0
Urinary retention	31	0	0	0
Hematuria	30	1	0	0

**Table 6.** The clinical data and risk factors for all cases

No.	Age	PS	FIGO stage	Histologic tumor grade	Pelvic lymph node metastasis	Maximal diameter of the primary tumor (cm)	Concurrent cisplatin chemotherapy	ADC mean × 10^−3^ mm^2^/s	Local control	Local control duration (M)
1	64	0	4a	moderate	positive	8.5	negative	0.987	control	31
2	60	0	2b	moderate	negative	4.3	positive	0.960	control	93
3	65	0	4a	moderate	negative	4.4	positive	0.892	recurrence	5
4	70	0	2b	moderate	negative	4.1	positive	0.100	control	71
5	82	0	3b	moderate	negative	3	positive	0.107	control	66
6	77	0	3b	poor	negative	4.6	positive	0.106	control	79
7	64	0	2b	well	negative	5.1	positive	0.965	control	71
8	57	0	1b1	moderate	negative	2.2	positive	0.692	control	63
9	66	0	3b	moderate	positive	5	positive	0.939	control	38
10	35	0	2b	moderate	positive	6	positive	0.942	control	28
11	62	0	3b	well	positive	6	positive	0.726	recurrence	10
12	63	0	2b	moderate	negative	3.5	positive	0.989	control	44
13	60	0	2b	poor	negative	4.8	positive	0.917	control	39
14	70	0	3b	moderate	positive	5	positive	0.828	control	41
15	44	0	2b	poor	positive	5.5	positive	0.980	control	40
16	62	0	2b	moderate	negative	5.8	positive	0.767	recurrence	21
17	35	0	2b	moderate	negative	5.4	positive	0.940	control	20
18	57	0	3b	poor	negative	5.5	positive	0.843	control	28
19	54	0	1b2	moderate	positive	3.7	positive	0.918	recurrence	6
20	72	0	2b	poor	negative	4.2	positive	0.894	recurrence	3
21	86	1	2b	moderate	negative	3.2	negative	0.831	recurrence	19
22	25	0	2b	moderate	positive	6.8	positive	0.906	control	21
23	57	0	1b1	poor	positive	2	positive	0.102	control	22
24	47	0	2b	moderate	positive	4.4	positive	0.805	recurrence	6
25	33	0	2b	poor	positive	6.1	positive	0.101	control	15
26	72	0	1b1	moderate	positive	5.3	negative	0.855	recurrence	7
27	85	1	2a1	moderate	negative	2.8	negative	0.947	control	16
28	79	2	4a	moderate	positive	5.9	negative	0.963	control	14
29	87	2	3a	moderate	negative	6	positive	0.100	control	8
30	59	0	2b	moderate	positive	4.7	positive	0.930	control	13
31	67	0	2a1	moderate	positive	4.2	positive	0.870	recurrence	5

ADC, apparent diffusion coefficient; FIGO, The International Federation of Gynecology and Obstetrics; PS, performance status.

## Discussion

In the present study, the average values of ADCmean for the primary tumor of the cervical cancer with the recurrence and non-recurrence were found to be 0.840 ± 0.064×10–3mm^2^/s and 0.949 ± 0.082×10–3 mm^2^/s, respectively. The difference in ADCmean between the two groups was statistically significant (*p* < 0.001). Additionally, the 2-year DFS probabilities for patients with cervical cancer of ADCmean ≥ 0.900×10–3 mm^2^/s and <0.900 ×10–3 mm^2^/s were 95% and 24%, respectively (Figure 3). The difference in the DFS probability between patients with cervical cancer of ADCmean ≥ 0.900×10–3 mm^2^/s and <0.900 ×10–3 mm^2^/s, calculated using the log–rank test, was statistically significant (*p* < 0.001).

Although RT is the optimal therapy for cervical cancer with an appreciable outcome, treatment for a tumor relapse remains tough. Thus, we consider it clinically essential to find patients with a high-risk for recurrence within a short time and who might benefit from additional or novel therapies, such as targeted agents with chemotherapy or adjuvant consolidation chemotherapy after RT.^20,21^ In previous studies, the stage, tumor size, histological type, histological grade, presence of lymphovascular space invasion and metastasis to regional lymph nodes at the time of treatment have been reported to be significant prognostic factors for cervical cancer.^22–24^ However, these parameters are not sufficient to accurately predict prognosis. It is challenging to predict the prognosis of patients treated with RT without performing histopathological retrieval. Therefore, additional markers would help determine a patient’s risk of recurrence or death. It is now accepted that new approaches for pre-treatment of cervical cancers are pivotal to further the disease’s favorable prognosis.

Quantitative assessment is possible by calculating the ADC, which is measured by DWI.^25^ It has been suspected that the decreased ADC values in malignant tumors may be caused by their increased tissue cellularity or cell density, larger nuclei with more abundant macromolecular proteins, and less extracellular space.^26–28^ Table 7 summarizes published reports of the risk factor for primary cervical cancer recurrence associated with ADC. A few previous studies have reported that DWI has the potential for predicting disease control or survival in cervical cancer patients treated with curative intent.^11,29–34^ Payne et al reported that the ADC values are expected to decrease when considering increasing tumor grades, as higher-grade tumors typically have a higher cellular density, resulting in restricted water diffusion in cervical cancer. Lower pre-treatment ADC values were associated with worse DFS in early-stage cervical cancer patients treated mostly with surgery.^11^ Regarding patients treated with RT, a previous study demonstrated that a lower pre-treatment 95th percentile ADC was associated with worse DFS.^34^ Ho et al found that pre-treatment ADC was an independent predictor of DFS in cervical cancer patients treated with RT.^32^ Onal et al demonstrated that pre-treatment ADC in cervical cancer patients treated with RT was an independent prognostic factor for DFS and OS.^29^ Although several values of ADC have been used for prognostic factors in cervical cancer, the complicated calculation methods were used for some factors. The method of calculation for prognostic factors should be possibly uncomplicated in daily clinical task. We consider that the measurement of ADCmean was uncomplicated method, and ADCmean was appropriate for prognostic factors in cervical cancer.

**Table 7.** The summary for published reports of the risk factor for primary cervical cancer recurrence associated with ADC

Firsrt Author	Pt No	Median follow-up	Timing of MRI	Histology	Endpoint	Prognostic factor	cut-off value	p
Nakamura K (36)	80pts	32.0M	Pretreatment	Squamous cell carcinoma, all	DFS	ADCmean	0.852 × 10^−3^ mm^2^/s	<0.001
						ADCmin	0.670 × 10^−3^ mm^2^/s	0.0210
Onal C (29)	44pts	25.0M	Pretreatment Posttreatment	Squamous cell carcinoma, all	DFS OS	ADCmean	DFS, 0.878 × 10^−3^ mm^2^/s	0.006
							OS, 0.878 × 10^−3^ mm^2^/s	0.006
Park JJ (31)	67pts	32.4M	Pretreatment During treatment	Squamous cell carcinoma, 59pts Non-Squamous cell carcinoma, 8pts	DFS	Pre - during treatment /pretreatment ADCmean x 100	35.1%	<0.001
Gu KW (33)	124pts	43.5M	Pretreatment Posttreatment	Squamous cell carcinoma, 103pts Adenocarcinoma/other, 21pts	DFS CSS OS	Post - pretreatment /pretreatment ADCmean x 100	DFS, 27.8%	0.001
							CSS, 16.1%	0.002
							OS, 16.1%	<0.001
Ho JC (32)	69pts	16.7M	Pretreatment	Squamous cell carcinoma, 48pts Adenocarcinoma/other, 21pts	DFS	ADCmean	0.940 × 10^−3^ mm^2^/s	0.02
Our study	31pts	25M	Pretreatment	Squamous cell carcinoma, all	DFS	ADCmean	0.900 × 10^−3^ mm^2^/s	<0.001

ADC, apparent diffusion coefficient; CSS, cancer Specific Survival; DFS, disease-free survival; OS, overall survival.

The most common histopathology subtype is squamous cell carcinoma, while adenocarcinoma is relatively rare.^35^ However, adenocarcinoma has the propensity to have a higher ADC than squamous cell carcinoma.^36^ Therefore, we assessed the ADC values exclusively in patients with squamous cell carcinoma. Although ADCmean was selected as a risk factor for the recurrence in this study, the other values of such as minimum and maximum values of ADC were used for risk factors in the other studies. Because the minimum or maximum values of ADC are measured as very low or high for hematoma, cystic or necrotic portions of cervical tumor, measurement errors can occur. Nakamura et al reported that the ADC mean of primary cervical cancer was an independent predictive factor for disease recurrence by multivariate analysis due to evaluating whether pre-treatment ADCmax, ADCmean, ADCmin on MRI predicted the risk group of recurrence.^37^ Therefore, we selected the ADC mean as a risk factor for recurrence.

We acknowledge that there are some limitations to our study. First, our study could not be free of measurement errors because ADC values were derived from manually drawn ROIs. Second, our study was a retrospective study in a single-center, with a relatively small patient population and a relatively short follow-up period. A larger number of patients and long-term follow-up would support the strength of our data, and further confirmation by a prospective trial could reinforce our findings.

## Conclusion

Our findings suggest that ADCmean values of the primary tumor could serve as an indicator for the risk of disease recurrence in patients with pre-treatment assessment of cervical cancer.
